# Real-time converter of intermittent-scanned glucose sensors to continuous glucose monitors with potential future applications for insulin delivery

**DOI:** 10.1016/j.ohx.2025.e00684

**Published:** 2025-08-19

**Authors:** Pablo S. Rivadeneira, Alejandro Mira

**Affiliations:** Universidad Nacional de Colombia, Facultad de Minas, Grupo GITA, Cra. 80#65-223, Colombia

**Keywords:** Glucose monitoring, Intermittent scanned sensors, Measurement accuracy, Automated insulin delivery systems

## Abstract

This paper presents the development of a transmitter that transforms intermittent glucose sensors (isCGM) into a continuous and real-time glucose monitoring system (c-rtCGM), a key component in automated insulin delivery systems. The transmitter enhances the capabilities of conventional intermittent sensors by leveraging Near Field Communication (NFC) technology to capture raw glucose value and automatically transmit it via Bluetooth Low Energy (BLE-Bluetooth 4.2 Dual-Mode) to a smart device every five minutes. A specialized glucose monitoring application converts the raw values to blood glucose by applying a calibration based on a static linear model and a capillary blood glucose measurement. The accuracy and performance of the c-rtCGM were validated through a study involving 37 participants with type 1 diabetes, demonstrating its reliability compared to commercial transmitters. Values reported by the c-rtCGM system compared with the isCGM monitor system resulted in an overall mean average relative difference (MARD) around 9%. During the trial, the c-rtCGM system achieved a data transmission success rate of 96%, and only 2316 connection failures were recorded from the 66525 total connection attempts, indicating a high level of communication stability. The transmitter battery life lasted an average of 6.5 days, showing that it is necessary to recharge only once for the duration of the sensor (14 days). The main advantages of this customized transmitter, in contrast with the commercial versions, are reliability, cost, and the flexibility of its software, since its processor (an ESP32) can be easily programmed to fulfill other helpful tasks in managing glucose levels with automated insulin delivery systems.

## Graphical abstract

**Figure:**
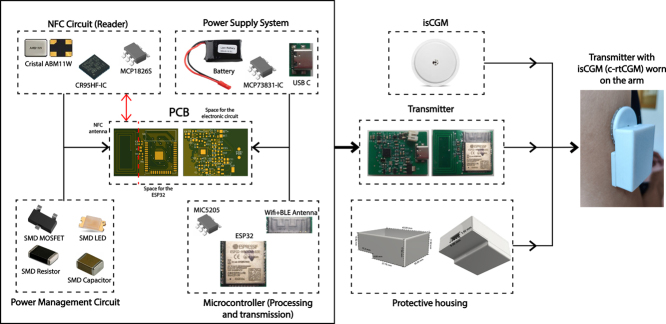

## 1 Specifications table

**Table:** 

**Hardware name**	Converter of intermittent-scanned glucose sensors for real-time monitoring (c-rtCGM).

**Subject area**	Control engineering and medicine.

**Hardware type**	Transmitter of intermittent-scanned glucose sensors

**Closest commercial analog**	MiaoMiao Transmitter

**Open source license**	Creative Commons Attribution-ShareAlike 4.0 International License (CC BY-SA 4.0)

**Cost of hardware**	$53.42

**Source file repository**	https://doi.org/10.17605/OSF.IO/4SGKC

## 2 Hardware in context

Type 1 diabetes mellitus (T1DM), which typically manifests during childhood, adolescence, or early adulthood, has an autoimmune origin that prevents the pancreas from producing insulin. As a result, glucose regulation is affected, and exogenous insulin administration is required. Since the 1970s, T1DM management has undergone a significant transformation due to advances in glucose monitoring technologies. Initially, self-monitoring blood glucose (SMBG) devices, commonly known as fingerstick meters, were introduced. These devices measure blood glucose from a drop of capillary blood and are typically used three to four times a day. However, their low sampling frequency limits the detection of significant fluctuations and critical episodes, such as hypoglycemia or hyperglycemia between measurements, as noted by Cappon et al. [1].

To overcome these limitations, more advanced devices have been developed, including continuous glucose monitors (CGMs) and intermittently scanned continuous glucose monitors (isCGMs). Both types use a minimally invasive needle inserted into subcutaneous tissue to detect an electrical signal generated by the enzymatic reaction of glucose oxidase, which is then converted into glucose concentration through a device-specific calibration process [1], [2]. Unlike SMBG, CGMs and isCGMs enable frequent monitoring over several consecutive days, generating time series that reveal trends and patterns in glycemic control [2], [3], [4]. Typically, CGMs provide automated readings every five minutes, while isCGMs require manual scanning to access the data [5].

According to the standard ISO 15197:2015, these glucose monitors must have an accuracy error of less than 15 mg/dL to be acceptable in clinical decision-making, with the Mean Absolute Relative Difference (MARD), which compares sensor readings to a reference standard, being the most common metric for evaluating accuracy. In Nagl et al. [6], the accuracy of three commercial devices is assessed: the CGMs Dexcom G6 (Dex) and Medtronic Enlite (MEL), and the isCGM FreeStyle Libre Sensor V1 (FSL), obtaining MARDs of 10.3%, 8.5%, and 13.3%, respectively. Eichenlaub et al. [7] repeated this comparison with newer versions, obtaining values of 9.6%, 16.1%, and 10.6%. Although accuracy decreases during hypoglycemic events (13% MARD), they are acceptable for clinical assessments, but not yet fully compliant with ISO 15197:2015.

Further studies have focused on the accuracy of FSL. Bailey et al. [8] reported a MARD of 11.4% in 72 participants, while Olfsdottir et al. [9] found a value of 13.2% in 58 adults, demonstrating that it is reliable for clinical assessment. Additional comparisons among sensors can be found in [10], [11]. Comprehensive reviews on CGM and isCGM technologies are available in [1], [4], [12], [13].

**Fig. 1:**
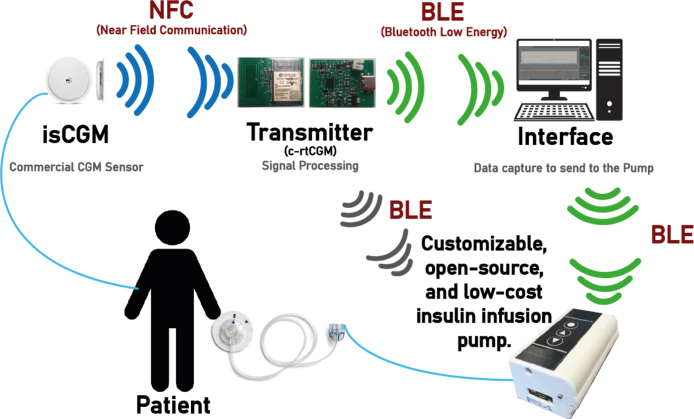
Overview of an automated insulin delivery system. The developed transmitter converts isCGM into c-rtCGM system. It retrieves a raw glucose value via NFC every 5 min and transmits it via BLE to a smart device, such as a computer, smartphone, or insulin pump. A dedicated application converts the raw value to blood glucose, and this measurement can be used to compute the required insulin dose and applied to a person if an insulin infusion pump is integrated.

A key advancement in T1DM management is the integration of CGMs with insulin pumps, as seen in systems as Dexcom and Medtronic, allowing for closed-loop control and improving automatic glucose regulation. In contrast, the FSL does not natively connect to insulin pumps. This sensor-pump combination forms the foundation of Automated Insulin Delivery Systems (AIDS), where an algorithm automatically calculates the appropriate insulin dose [14], as illustrated in Fig. 1.

Although the FSL does not provide real-time readings, it is notable for its low cost (one-third to one-half the price of other CGMs) and long lifespan (14 days, compared to 10 days for Dex and 6 days for MEL) [5]. To address the FSL manual scanning limitation, third-party solutions, such as the MiaoMiao (MM) transmitter, have emerged. This device attaches to the FSL and continuously transmits data via BLE, eliminating the need for manual scanning, converting the FSL into a fully CGM [15], [16], [17]. It acts as an external reader, accessing data wirelessly using an NFC protocol without compromising data integrity.

Various studies have documented clinical benefits of the FSL + MM combination, including reduced fear of hypoglycemia and improvements in metabolic control and quality of life [15], [16]. For instance, Elbalshy et al. [16] reported significant improvements in patients’ perceived safety, and Kublin et al. [15] documented statistically significant reductions in glycated hemoglobin (HbA1c) levels, from 7.42% to 6.72% in adults and from 7.03% to 6.03% in children (P<0.001). They also reported a substantial decrease in weekly hypoglycemic episodes and an improved quality of life, without incidents of severe hypoglycemia or ketoacidosis, in adults. These findings underscore the effectiveness of transmitters as valuable tools for enhancing diabetes monitoring and management.

The development of these transmitters has also enabled the emergence of several applications for CGM data visualization and analysis using FSL, including Glimp and Tomato, as well as open-source tools like Spike and xDrip+, which is widely used among individuals with T1DM [16], [18]. Additionally, apps like AndroidAPS, OpenAPS, and Loop can be connected to insulin pumps to create insulin delivery systems commonly known as “do-it-yourself” artificial pancreas systems [19], [20].

Although the aforementioned open-source solutions have democratized access to advanced technologies for closed-loop glucose regulation, they often lack interoperability, robust cybersecurity measures, and do not permit the integration with other customized hardware or software systems. Applications such as xDrip+, Spike, or Nightscout may not always include encryption or data traceability, which raises concerns about privacy and compliance with regulations.

**Fig. 2:**
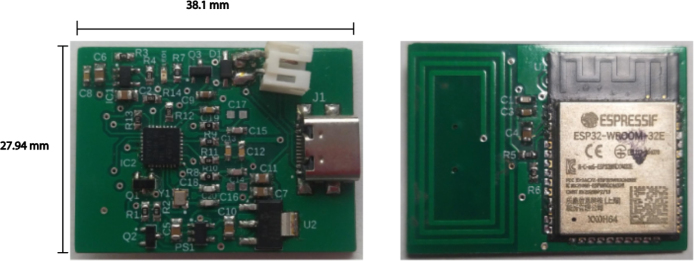
Final electronic circuit printed as PCB without casing. Top (left) and bottom (right) views of the custom-developed transmitter PCB, measuring 27.94 mm × 38.1mm.

In this context, the transmitter developed in this work (see Fig. 2) is presented as a portable alternative that transforms the FSL sensor into a real-time continuous glucose monitor (c-rtCGM). This device retrieves data every five minutes using NFC and automatically transmits it via BLE to a smart device, eliminating the need for human intervention. Unlike MM, its software is easily configurable and enables additional tasks that MM does not support. For example, advanced security protocols can be added to the data transmission, and it can be configured to activate or deactivate a connected insulin pump based on glucose predictions using a mathematical model and actual measurements. This can significantly reduce energy consumption, as demonstrated in a recent article by Goez and Rivadeneira [21].

Furthermore, the transmitter enables direct data transmission to the cloud for backup or sharing purposes, thanks to its ESP32-based architecture with WiFi connectivity. Its design also allows adaptation to other devices compatible with ISO/IEC 15693, ISO/IEC 14443-3, and ISO/IEC 18000-3M-1 communication protocols via the CR95HF-IC chip. However, this option has not been explored, as our primary motivation is the development of low-cost systems for diabetes applications.

## 3 Hardware description

**Fig. 3:**
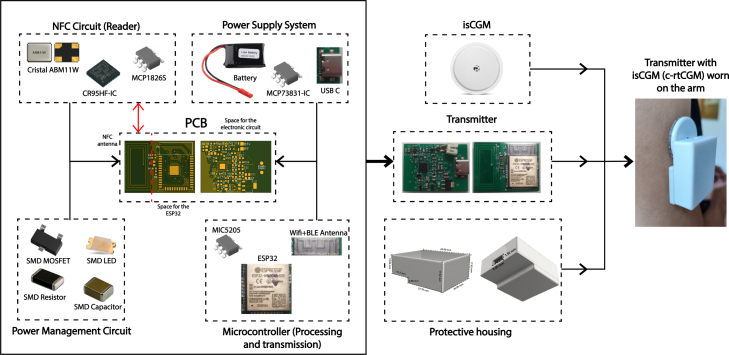
This diagram illustrates an overview of the architecture of the transmitter system designed for continuous glucose monitoring. The system comprises several essential components, each playing a crucial role in its operation and final building.

The device has been carefully designed to ensure smooth and efficient communication between an isCGM and a smart device (mobile, computer, or insulin pump) utilizing NFC and BLE (Bluetooth 4.2 Dual-Mode. It supports the Bluetooth Classic (BR/EDR) as the BLE.). The system is composed of several key modules interacting to optimize its operation and reduce energy consumption, as is depicted in Fig. 3.

The diagram illustrates how each component is connected and highlights its specific function within the system. At the core of the system is the NFC circuit, which includes an NFC antenna and the CR95HF-IC integrated circuit, enabling seamless communication with the CGM sensor. The power supply system consists of a LiPo battery, an MCP73831 charger, and a USB-C input, ensuring a stable and reliable energy source. Additionally, a power management circuit regulates voltage and protects the system from potential spikes using MOSFETs, resistors, capacitors, and LEDs. For data processing and wireless transmission, the system integrates an ESP32 microcontroller, which features built-in WiFi and BLE, facilitating real-time connectivity. All components are assembled on a custom-designed printed circuit board (PCB), which is responsible for acquiring data from the CGM sensor and transmitting it wirelessly. Lastly, the protective housing encloses the entire system, safeguarding its components while enabling secure attachment to the CGM sensor.

### 3.1 NFC communication

The CR95HF-IC, a controller that manages NFC communications, converts NFC data to serial. This chip acts as a key intermediary between the isCGM and the rest of the transmitter, decoding NFC frames and transmitting the data to the microcontroller via the SPI serial interface. This process is essential for the system to interpret the glucose level data measured by the sensor correctly.

The CR95HF-IC employs the SPI protocol under the ISO/IEC 15693 standard for serial communication with a microcontroller as the ESP32.

The NFC circuit is equipped with a 27.12 MHz crystal that provides the necessary clock frequency for the CR95HF-IC to function properly. This component ensures the correct processing of NFC signals and maintains precise synchronization for continuous data exchange.

Another critical element in this subsystem is the MIC5205 regulator, which provides a stable power supply to the NFC circuit. This minimizes voltage fluctuations that could compromise data transmission. Additionally, the MIC5205 offers low-noise regulation, which is essential to avoid interference during NFC reading. Thus, it ensures optimal performance of the CR95HF-IC, particularly in portable medical device applications where precision and accuracy are vital.

Finally, the NFC antenna, directly integrated into the transmitter’s PCB, is designed to operate at the standard frequency of 13.56 MHz. This allows reliable data reading from the FreeStyle Libre sensor, even under low-energy conditions. The antenna is optimized to provide high sensitivity and precision, enabling the CR95HF-IC to detect and process NFC signals efficiently and ensure continuous and stable transmission of glucose data.

### 3.2 ESP32 WEMOS D1 mini module

The ESP32 WEMOS D1 Mini acts as the brain of the transmitter, handling BLE connectivity and the system’s control logic. This design leverages only the core of this board, allowing for a more efficient and compact approach. This microcontroller is ideal due to its energy-efficient features, including its ability to enter *Deep Sleep* mode, which consumes minimal power (around 20μA) to prolong battery life.

Below are the pins involved in the ESP32 configuration for SPI:

• **Serial Clock (SCK - IO18)**: Controls the system clock signal.
• **Master In Slave Out (MISO - IO19)**: Allows the slave to send data to the master.
• **Master Out Slave In (MOSI - IO23)**: Sends data from the master to the slave.
• **Chip Select (CS0 - D05)**: Activates or deactivates the data-receiving peripheral, also known as *Slave Select* (SS) in other architectures.
• **Interrupt Request (IRQ - IO26)**: Hardware interrupt line used for configuring the CR95HF-IC. Although there is no dedicated pin, IO26 is selected due to its proximity to the SPI pins.

This pin distribution ensures effective communication between the ESP32 and the CR95HF-IC, allowing efficient use of the SPI protocol.

When the system needs to perform an NFC sensor reading, the ESP32 wakes up from sleep mode and, through a digital signal, enables the MIC5205 regulator, powering the NFC circuit to read data from the FSL. After transmitting the data via BLE, the ESP32 automatically returns to *Deep-Sleep* mode, and the NFC regulator is turned off, thus reducing power consumption.

The ESP32 manages the transmitter’s power and handles BLE communication, enabling the transmission of glucose data to a mobile device or monitoring application. Thanks to its connectivity capabilities and programming flexibility, the ESP32 is ideal for this type of portable medical application.

### 3.3 Power regulation and management

To maintain system stability, the design incorporates several voltage regulators that ensure an adequate power supply for the main components. Among them are the MIC5205, which regulates power to the NFC circuit, and the MCP1826S, which provides a stable voltage source for other system modules, such as the ESP32.

These regulators are accompanied by a set of decoupling capacitors and filters, which play a fundamental role in reducing noise in the power supply to the components, improving the device’s overall stability, and preventing interference that could affect its performance.

The device is powered by a 600 mA LiPo battery, which, thanks to the implemented power-saving mode, allows continuous operation for more than 15 h on a single charge. The ESP32 module operates in low-power modes and manages the selective activation of the NFC circuit only when necessary.

The MCP73831-IC, an efficient controller that ensures fast and safe battery recharging, controls the battery charging process. The estimated charging time is between 3 and 3.5 h, and an LED indicates the battery status during the process, facilitating its monitoring.

### 3.4 PCB and housing design

**Fig. 4:**
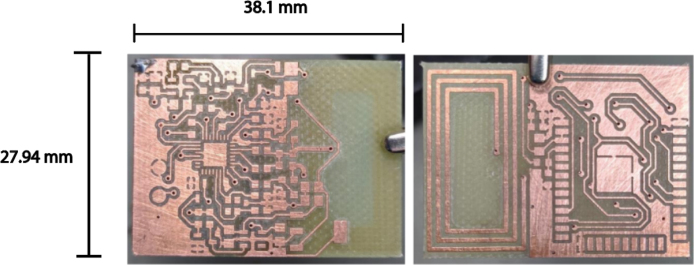
Top (left) and bottom (right) views of the two-layer PCB used for the transmitter, measuring 27.94 mm × 38.1mm. The image on the right shows the integrated NFC antenna, enabling wireless communication with the CMG sensor. The design includes power regulation and the ESP32 microcontroller core.

The device is built on a two-layer PCB, allowing a compact integration of all electronic components. An image of the PCB used for assembling the transmitter is shown in Fig. 4.

The NFC antenna is integrated into the PCB and is designed to operate at the RF frequency of 13.56 MHz, thus ensuring reliable communication. Pertinent measurements have been conducted to guarantee that the power circuits are correctly sized, contributing to stable and efficient system operation.

**Fig. 5:**
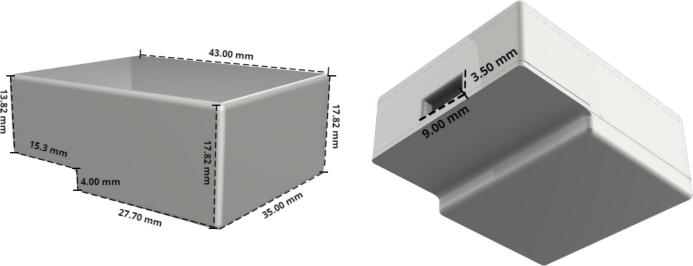
3D-printed protective housing for the transmitter, shown from multiple angles with annotated dimensions. The enclosure safeguards the internal electronics while maintaining a compact form factor.

In Fig. 5, the housing of the transmitter is shown, designed to protect the electronic components. Since this is an experimental transmitter, the case was 3D printed using standard polylactic acid (PLA). However, if its use is intended in other contexts, and more frequently, it is recommended to use medical-grade PLA (for instance, PLA biocompatible ISO 10993-5/10 certified), a skin-safe material validated for prolonged human contact.

The figures provide the housing dimensions, ensuring it is as compact as possible while still accommodating and protecting all electronic components. Additionally, the design allows the device’s battery to be recharged without needing to remove the housing.

**Fig. 6:**
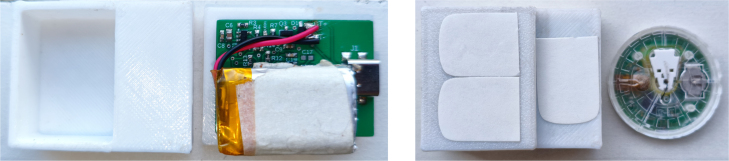
In the left image, the unassembled casing is shown alongside the transmitter with its battery connected. In the right image, the transmitter is fully assembled and secured with hypoallergenic adhesive tape to the isCGM, ensuring proper attachment.

In Fig. 6, the integration of the sensor, battery, and transmitter, along with the arrangement of their components, is illustrated. Additionally, hypoallergenic double-sided adhesive tapes are incorporated to secure the transmitter to the sensor. These tapes also adhere to the patient’s skin, providing a secure contact to avoid skin irritation.

**Fig. 7:**
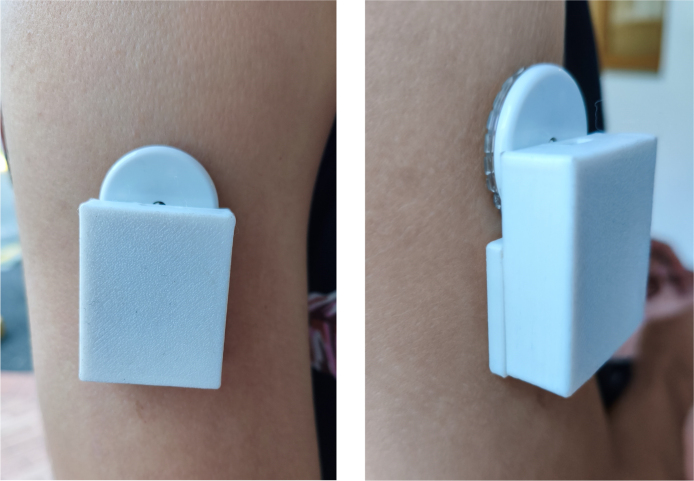
In the left image, a front view of the transmitter attached to the FSL on the skin is shown. In the right, an angled view of the device is presented, highlighting its fixation and position on the patient’s arm.

In Fig. 7, the transmitter’s integration with the CGM sensor on the patient’s skin is illustrated. The design ensures that the device remains securely in place during daily activities, providing uninterrupted glucose monitoring. Also, an elastic band can be used to hold the transmitter if necessary.

### 3.5 Software description

This section will discuss in detail the most relevant aspects of the C++ code developed to run on the transmitter, specifically for the Wemos D1 Mini ESP-32S.

To correctly manage SPI (Serial Peripheral Interface) communication between the Wemos D1 Mini ESP-32S and the NFC reader, a custom structure is implemented that controls locking and mutexes. This structure facilitates efficient access to shared resources during SPI transactions.

**Figure:**
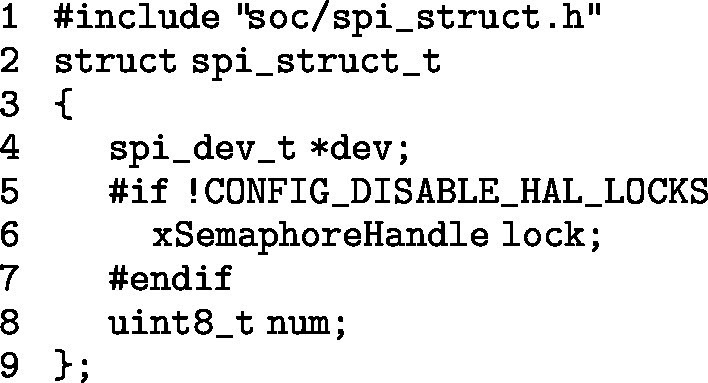

The spi_struct_t structure ensures effective synchronization between SPI operations, improving performance and avoiding conflicts in accessing shared resources. During the initialization phase of the program, the SPI bus is configured to ensure smooth communication between the master device (Wemos D1 Mini ESP-32S) and the slave (NFC reader). This is achieved by adjusting the SPI bus controller.

**Figure:**
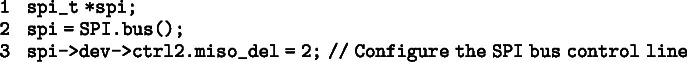

This code configures the SPI bus control line to ensure that data is exchanged efficiently from the start of the system, optimizing communication between the devices.

The overall operation of the code can be broken down into the following steps:

1. **NFC Data Reading**: The function float ReadMemoryCommand() is implemented to read the memory blocks of the NFC reader. This function retrieves the blood glucose concentration value, directly interacting with the NFC reader hardware to extract the necessary data.
2. **Bluetooth Connection**: The system waits for a Bluetooth connection with a receiving device. To check for the presence of an NFC device within range, the function bool InventoryCommand() is used. Subsequently, the function bool SetProtocolCommand() sets the appropriate NFC protocol according to the available reader, ensuring efficient communication.
3. **Pairing and Data Transmission**: Once the Bluetooth connection is established, the device pairs with the receiver. If pairing is successfully completed, the glucose data obtained from the NFC reader is sent to the receiver. If pairing is not achieved after 20 s, the device enters low-power mode (sleep).
4. **Sleep Mode**: The function void goToSleep(int timeToSleep) puts the Wemos D1 Mini ESP-32S in low-power mode for a defined period (in this case, 5 min). After this period, the system reactivates and restarts the process of reading and sending data.
5. **Error Management and NFC Reset**: To ensure continuity in data reading, the function void CR95HF Reset() resets the NFC device in case of communication failures. This ensures that the system is always ready to perform new readings and send data to the receiver.

To send data to the receiver via BLE, a service is created on the Wemos D1 Mini ESP-32S to handle this task. In void setup(), the following lines are executed to properly initialize the BLE service, allowing efficient communication with mobile devices or compatible receivers.

**Figure:**
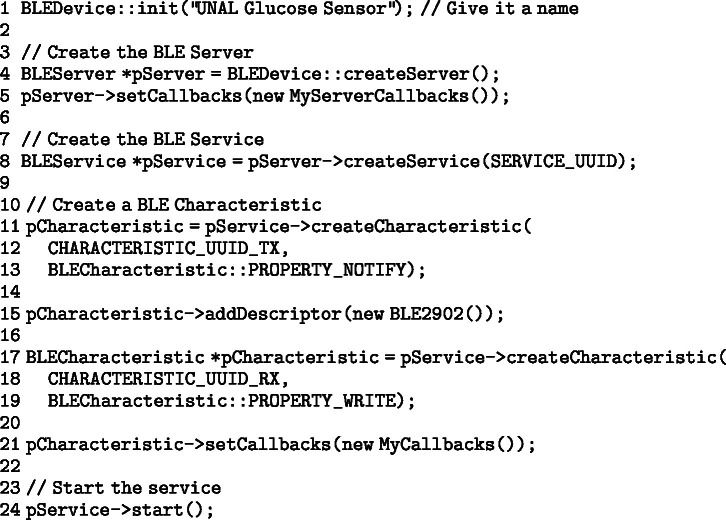

It is important to note that the system has a waiting cycle of approximately 5 min between readings. In case of connection errors, this indicates that the Wemos D1 Mini ESP-32S was not fully ready to execute the query. Therefore, the system provides a robust and efficient solution for glucose data capture and transmission, emphasizing the reliability of communication between the various hardware and software modules.

Due to the modularity of the development, the code cannot be presented conventionally. However, it can be explored through a link available in the OSF repository (Link).

### 3.6 Utility of the hardware for conventional and innovative laboratory tasks

• The transmitter enables the conversion of intermittent readings from sensors such as the FreeStyle Libre into real-time glucose data, facilitating accurate glycemic trend analysis in metabolic studies without relying on commercial continuous glucose monitoring (CGM) systems.
• Its open-source firmware, built on the ESP32 platform, can be adapted for biomedical applications requiring real-time data acquisition and wireless communication via BLE/NFC, particularly in low-cost or resource-constrained environments.
• The device’s modular architecture allows seamless integration with custom control loops, such as model predictive controllers (MPC), serving as an experimental platform for validating closed-loop drug delivery systems.
• Due to its firmware and hardware flexibility, the transmitter can be repurposed for laboratory automation tasks involving periodic data retrieval from NFC-compatible devices, including biosensors, analytical instruments, or wearable systems used in human or animal research protocols.
• BLE and WiFi connectivity enable integration into Internet of Things (IoT) frameworks focused on remote monitoring, continuous data logging, and synchronization with cloud-based analytics or supervisory control platforms.

## 4 Design files summary

**Table:** 

Design filename	File type	Open source license	Location of the file
Cover_v1.f3d	3D Design File (Fusion 360)	Creative Commons Attribution 4.0 International	Available with the article ( Repository )

Circuit.brd	PCB Layout File (Eagle)	CERN Open Hardware License v2	Available with the article ( Repository )

Schematic_card.sch	Circuit Schematic File (Eagle)	CERN Open Hardware License v2	Available with the article ( Repository )

BM019.pdf	NFC Antenna Datasheet	Public Domain	Available with the article ( Repository )

CR95hf.pdf	NFC Controller Datasheet	Public Domain	Available with the article ( Repository )

esp32-wroom-32d_esp32-wroom-32u.pdf	ESP32 Module Datasheet	Public Domain	Available with the article ( Repository )

## 5 Bill of materials summary

**Table:** 

Designator	Component	Number	Cost per unit - currency	Total cost - currency	Source of materials	Material type
M1	ESP32 WROOM Module	1	$2.68	$2.68	Link	Semiconductor

M2	CR95HF-IC (NFC) Package VFQFPN32 (5 × 5 mm)	1	$3.69	$3.69	Link	Semiconductor

M3	Crystal ABM11W - 27.12 MHz	1	$0.80	$0.80	Link	Inorganic

C1	SMD 0603 Capacitor 220 pF/16 V	2	$1.43	$2.86	Link	Ceramic

C2	SMD 0603 Capacitor 150 pF/16 V	2	$0.81	$1.62	Link	Ceramic

C3	SMD 0603 Capacitor 15 pF/16 V	2	$0.81	$1.62	Link	Ceramic

C4	SMD 0603 Capacitor 1000 pF/50 V	1	$0.1	$0.1	Link	Ceramic

C5	SMD 0603 Capacitor 0.1 uF/50 V	3	$0.14	$0.42	Link	Ceramic

C6	SMD 0805 Capacitor 4.7 uF/50 V	7	$0.48	$3.36	Link	Ceramic

D1	1N5819 Diode SOD-123 Package	1	$0.25	$0.25	Link	Semi-conductor

IC1	MCP73831T-3ACI/OT	1	$0.76	$0.76	Link	Semi-conductor

LED1	0603 Green LED	1	$0.51	$0.51	Link	Semi-conductor

IC2	MIC5205 Voltage Regulator, SOT23-5 Package	1	$0.54	$0.54	Link	Semi-conductor

IC3	MCP1826S Voltage Regulator, SOT223 Package	1	$0.91	$0.91	Link	Semi-conductor

T1	2N7002 Transistor, SOT23 Package	2	$0.1	$0.2	Link	Semi-conductor

T2	PMV250 Transistor, SOT23-3 Package	1	$0.24	$0.24	Link	Semi-conductor

R1	10 kΩ SMD Resistor 0603	4	$0.68	$2.72	Link	Passive

R2	470 Ω SMD Resistor 0603	1	$0.4	$0.4	Link	Passive

R3	2 kΩ SMD Resistor 0603	1	$0.4	$0.4	Link	Passive

R4	27 kΩ SMD Resistor 0603	1	$0.48	$0.48	Link	Passive

R5	270 Ω SMD Resistor 0603	1	$0.39	$0.39	Link	Passive

R6	0.0 Ω SMD Resistor 1206	2	$0.44	$0.88	Link	Passive

R7	330 Ω SMD Resistor 0603	2	$0.14	$0.28	Link	Passive

R8	3.3 kΩ SMD Resistor 0603	2	$0.57	$1.14	Link	Passive

B1	500mA LiPo Battery	1	$5.95	$5.95	Link	Other

P1	USB Type-C charging port	1	$1.17	$1.17	Link	Other

PCB	Approximate PCB manufacturing cost	1	$5.44	$5.44		Composite

Casing	Casing made of PLA	1	$10	$10		Polymer

**TOTAL AMOUNT**	**Rounded value**			**$53.42**		

## 6 Build instructions

**Fig. 8:**
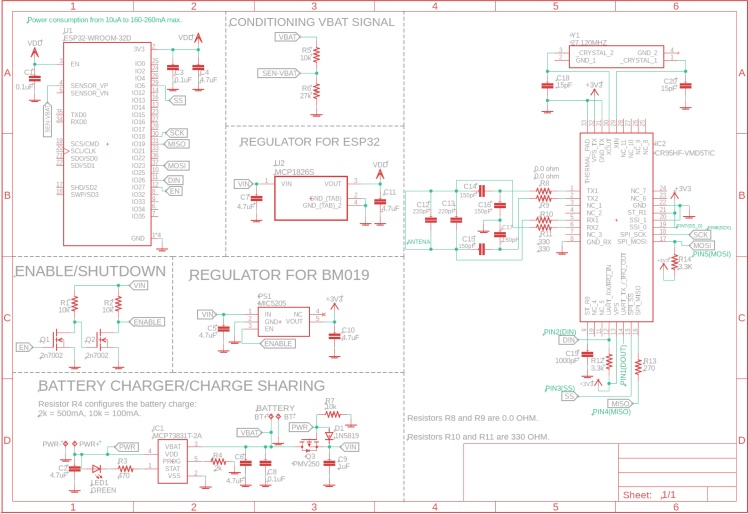
Detailed schematic of the transmitter’s electronics, illustrating the battery charging circuit, power regulation for both the ESP32 and the NFC module, and the signal lines that enable glucose data capture and BLE transmission. The diagram highlights the CR95HF-IC NFC interface, the battery management system, and the main microcontroller connections.

To build the NFC-to-BLE transmitter, it was necessary to acquire the previously described materials in the Table in Bill of materials summary, which were soldered onto the PCB following the provided connection diagram of Fig. 8.

First, the connection diagram of the transmitter’s electronic components is presented in Fig. 8, including the power regulation system, the ESP32 core connection, the antenna arrangement, and the placement of each element in the system.

**Fig. 9:**
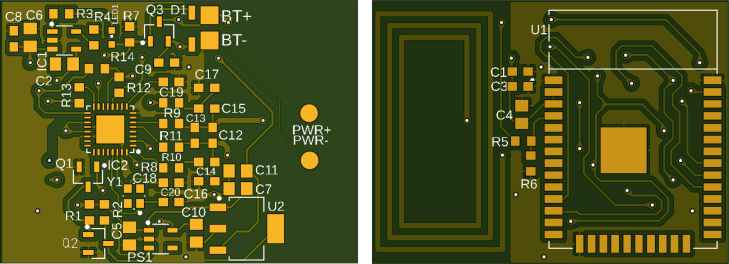
Top (left) and bottom (right) layers of the transmitter’s PCB, depicting the arrangement of SMD components (resistors, capacitors, diodes, and ICs) and the integrated NFC antenna. This connection diagram highlights how the ESP32, power regulators, and NFC module are placed and interconnected.

In Fig. 9, the printed PCB is presented, where the various components were soldered. The numbering of the elements (capacitors, resistors, diodes) and the location of the ESP32 core can be identified.

Once the transmitter components were soldered onto the PCB (it looks as Fig. 2), the housing was printed using the file carcasa.Gcode and maintaining the 3D printer with an 80% infill in PLA material, with a nozzle diameter of 0.3 mm, an extruder temperature of 250 °C, and a bed temperature of 100 °C.

## 7 Operation instructions

The transmitter captures data through the NFC reader and then transmits it either to a smart device (such as a computer or smartphone) or directly to an insulin pump via BLE (Bluetooth 4.2 Dual-Mode.). This section explains the process of transmitting data to the customized blood glucose monitoring user interface.

### 7.1 Device operation steps

Before beginning data collection, the developed application must request the necessary permissions to access the smart device’s Bluetooth services. This enables the Bluetooth connection and access to related functionalities. In an Android device:

**Figure:**
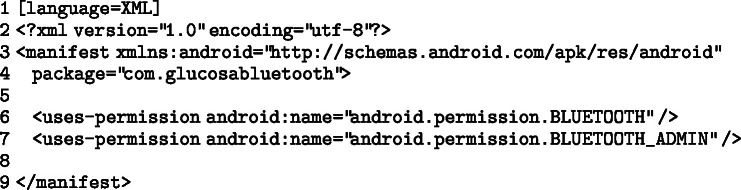

In a Windows device:

**Figure:**
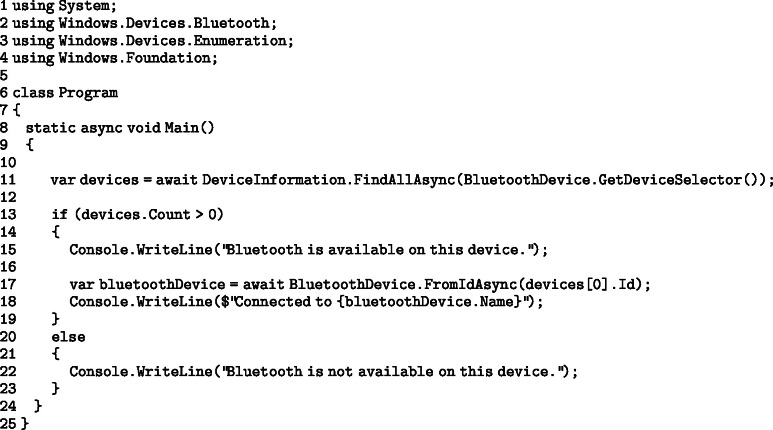

**Fig. 10:**
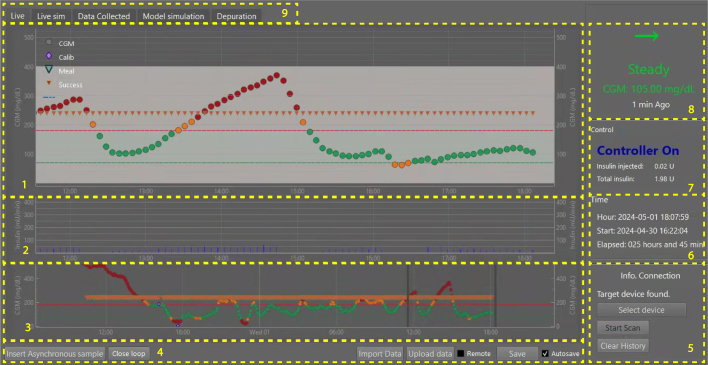
User interface for data visualization. This HMI permits monitoring a person’s glucose in an open or closed loop while receiving the data via Bluetooth.

Once the necessary permissions are granted, the next step is to establish a connection with the Bluetooth module, which in this case is the ESP32. The application provides a graphical interface where the user can select the transmitter, as illustrated in Fig. 10.

When the Bluetooth connection is successfully established, the application is ready to receive data. The information flows from the reader through the ESP32 module to the smart device. At this stage, it is crucial to correctly interpret the transmitted data. To achieve this, data processing and reception are managed through a Python-based program, which implements the necessary methods to read the information sent via Bluetooth and display it accurately in the user interface.

Once the data is received and interpreted, it is presented in a graphical environment designed to provide the user with a clear and accessible visualization. The user interface (UI), illustrated in Fig. 10, follows the human machine interface (HMI) philosophy, emphasizing reliability, redundancy, open architecture, communication, ease of installation, and usability. These principles ensure that users can efficiently access information with a single click, utilizing visual elements that enhance data interpretation on-screen. This version was envisioned to also integrate an insulin pump and can be used in manual mode, where the insulin dose is entered by the user, or in an automated closed loop, where algorithms are computing the insulin dose and communicating with the transmitter and pump.

The interface shown in Fig. 10 includes a blood glucose logging module that represents measurements using color-coded circles based on the recorded level: **Green:** Values between 70 and 180 mg/dL, **Yellow:** Measurements between 50-70 mg/dL and 180-220 mg/dL, and **Red:** Values below 50 mg/dL or above 220 mg/dL.

Additionally, orange triangles indicate successful data reception every five minutes, while purple diamonds log asynchronous measurements from the glucometer or CGM sensor. Green triangles represent carbohydrate intake. The interface panels provide various key functionalities for data monitoring and management, represented by boxes 1 to 9, where the box (1) shows the glucose time evolution (mg/dl), some boundaries, glucometer asynchronous display (Calib), carbohydrate intake (Meal), and successful glycemic transmission (Success), box (2) shows the administered insulin, box (3) shows the entire glucose evolution during the interface operation, and also defines an adjustable time window for sections 1 and 2, in box (4) are the data settings, import/upload/save data and manual glucose samples, box (5) describes the transmitter status, box (6) the elapsed time log, box (7) shows the open or closed loop mode and the total and previous insulin doses during the interface operation, box (8) shows the current glycemia value and its trend, and box (9) is the real-time recording, stored data, and system log.

The interface establishes a connection with the transmitter to collect data at five-minute intervals. Otherwise, the system will attempt to reconnect every minute until communication is restored. Additionally, it enables retrieving missing data in 15 min intervals, with a maximum storage capacity of eight hours in the sensor. For further analysis, data can be exported or imported in CSV format. A full description of the interface can be found in Goez and Rivadeneira [22].

After developing the application, performance tests were conducted to verify its functionality and reliability. These tests evaluated the application’s response under different conditions. For example, if the sensor was out of reading range, the application displayed a value of 0.0, indicating that the connection was not established correctly or that the sensor was not transmitting valid data. Conversely, if the sensor was within reading range, the application showed the current captured value.

### 7.2 Device calibration

The calibration process is essential for converting the raw signal obtained by the sensor into accurate blood glucose values. Since FSL measures glucose concentration in interstitial fluid rather than directly in the blood, an adjustment is necessary to correlate these measurements with actual blood glucose levels.

Calibration may vary from patient to patient, as the sensor’s response to interstitial glucose is influenced by individual factors such as tissue perfusion, metabolism, and glucose absorption delay. However, standardized calibration models have been developed to adjust sensor readings based on reference measurements obtained with a conventional glucometer. In Goez and Rivadeneira [22], it was found that a linear model of the form $G=\frac{CGM_{RAW}}{a}-b$ was enough to convert to blood glucose with high accuracy, where G represents the corrected blood glucose value, $CGM_{RAW}$ is the raw sensor reading, and a and b are experimentally determined adjustment parameters. These parameters are periodically recalculated using reference measurements to minimize deviations between sensor readings and actual blood glucose levels.

The calibration process consists of the following steps: (i) Reference measurement: Blood glucose is measured using a glucometer, and the value is recorded, (ii) Comparison with sensor reading: The glucose reading provided by the sensor is checked at the same moment, (iii) Application of the adjustment model: A calibration model, typically linear (described above) is applied, and (iv) Updating the transmitter algorithm: The system interface stores the new calibration and adjusts future sensor readings based on the obtained reference values. The calibration process must be repeated frequently at regular intervals or when significant deviations in the data are detected.

The calibration frequency has a significant impact on system accuracy. Experimental data analyzed in Goez and Rivadeneira [22] indicate that more frequent calibration improves measurement precision. The analyzed experimental results show that without calibration, the MARD is 8.68%, with calibrations every 24 h, the error reduces to 6.68%, and with calibrations every hour, the error is reduced to 2.41%.

These results suggest frequent calibration is essential for obtaining more reliable measurements and minimizing errors according to the ISO 15197:2015. However, high calibration frequency can be annoying for patients. Therefore, at least a daily calibration is suggested to maintain an acceptable MARD and increase adherence to glucose monitoring.

## 8 Validation and characterization

A comparative and observational single-center reading study was developed using the c-rtCGM developed here and the FSL sensor. The primary outcome was a quantitative evaluation of the c-rtCGM’s accuracy and clinical precision relative to the FSL. The secondary outcome was the performance of the hardware, specifically the rate of data transmission and the total duration of battery life.

The device was tested over a four-week period with 37 participants with type 1 diabetes. Both the designed transmitter (c-rtCGM) and the FSL were used in two stages: two weeks with an initial single calibration (uncalibrated) and two weeks with daily calibration (calibrated). The accuracy and performance assessment will include the MARD, the consensus error grid analysis, the rate of data transmission success, and the final duration of battery life.

The devices used in the study were: (i) the FSL sensor (version 1), which is factory-calibrated. It requires a one-hour warm-up period and has a usage duration of 14 days. This sensor detects glucose levels in the 40 to 500 mg/dL range. After scanning, the user can view the current glucose level, a trend arrow, and readings from the past eight hours, (ii) the designed Transmitter: as it is illustrated in Fig. 3, (iii) a smartphone Kalley Element Play with a SIM card, Bluetooth, and a light version of the data manager app to register data but blinded to the patients, and (iv) a PC running Windows 10 was used to connect to the phone, store the data, and to remotely monitor patients using the full version of the interface of Fig. 10.

### 8.1 Study independence and ethics

None of the device manufacturers involved participated in conducting this study.

The study was approved by the Ethics Committee of the Pablo Tobon Uribe Hospital, Medellin, Colombia (Ref. 2023/0.70). All patients provided informed consent, and their privacy was protected by restricting identification to the physician, while the remaining authors accessed a de-identified database.

### 8.2 Study design and participants

The participants were adults with type 1 diabetes, diagnosed at least one year before the study, and receiving treatment via continuous subcutaneous insulin infusion (CSII) or multiple daily insulin injections (MDI), were included. Pregnant women and patients with liver failure or chronic kidney disease with a glomerular filtration rate below 30 mL/min were excluded. Other considerations were taken as in Villa-Tamayo et al. [23].

Participants received training on using the FSL glucose monitoring system and were instructed to scan the FSL and manually record the glucose value from the FSL reader if they could not obtain a reading from the c-rtCGM. The initial and daily calibration were conducted based on the value provided by the FSL reader using the fingerstick measurement.

Glucose monitoring system calibration was performed by entering the glucose value into our customized app. Additionally, participants were blind to the information generated with the app to avoid altering their usual treatment during the study period.

### 8.3 Results

Thirty-seven subjects completed the study. The glucose outcome metrics are found in Table 1. Each participant used the c-rtCGM for a total of 28 days: 14 days without calibrations (only the initial one) and an additional 14 days with the instruction to perform daily calibrations. The MARD results between the FSL and the c-rtCGM, both with and without calibrations, are presented in Table 1.

The accuracy across different glucose ranges is detailed in Table 2. For both devices (calibrated and non-calibrated), the least similarity between the sensors occurred for glucose values <70mg/dL.

**Table 1:** Blood glucose results in different BG ranges, comparing the FSL and c-rtCGM with and without calibration. Statistics are included that reflect the accuracy of each calibration frequency.

	Calibrated		Uncalibrated	
	FSL	c-rtCGM	FSL	c-rtCGM
**Mean BG (mg/dL)**	168.195 ± 43.72	167.07 ± 41.36	167.235 ± 50.76	174.12 ± 53.225
**SD of BG (mg/dL)**	58.315 ± 27.50	60.59 ± 28.13	56.905 ± 28.235	65.675 ± 31.155
**Coefficient of Variation (%)**	33.755 ± 12.49	35.47 ± 13.65	32.685 ± 12.98	37.15 ± 14.565
**Percentage of time in range (%)**				
BG <54mg/dL (%)	0.245 ± 0.66	0.585 ± 1.21	0.245 ± 0.795	1.89 ± 2.66
BG <70mg/dL (%)	2.485 ± 4.27	5.27 ± 6.225	2.195 ± 4.63	4.385 ± 6.74
BG 70–180mg/dL (%)	62.445 ± 19.49	61.035 ± 24.4	64.54 ± 24.405	57.61 ± 25.185
BG >180mg/dL (%)	34.345 ± 19.19	33.27 ± 23.29	32.445 ± 24.36	37.19 ± 25.18
BG >250mg/dL (%)	18.60 ± 16.21	18.38 ± 18.425	17.9 ± 17.235	21.905 ± 20.64
BG >300mg/dL (%)	4.88 ± 11.55	4.875 ± 14.01	8.13 ± 16.975	9.66 ± 19.115

For the calibrated devices, the best accuracy was observed for BG>300 mg/dl, and when the FreeStyle Libre reported glucose values between 180 and 250 mg/dL, was 8.18%. For the non-calibrated devices, the best MARD was 9.19% for BG>300 mg/dl.

**Table 2:** MARD for different blood glucose (BG, mg/dL) ranges and rates of change (ROC, mg/dL/min), comparing calibrated and uncalibrated measurements from the developed transmitter and the FSL monitoring system.

BG Range (mg/dL)	MARD (%)	
	Calibrated	Uncalibrated
Overall	8.79	12.18
BG < 70	11.83	13.38
BG 70–180	8.47	11.81
BG 180–250	8.18	12.32
BG 250–300	7.96	14.19
BG > 300	7.54	9.19
ROC (mg/dL/min)		

	Calibrated	Uncalibrated

ROC < –2	10.84	19.2
ROC [–2, –1)	9.52	16.3
ROC [–1, 0)	8.90	15.37
ROC [0, 1]	8.21	14.53
ROC (1, 2]	7.96	14.30
ROC > 2	13.34	13.97

**Fig. 11:**
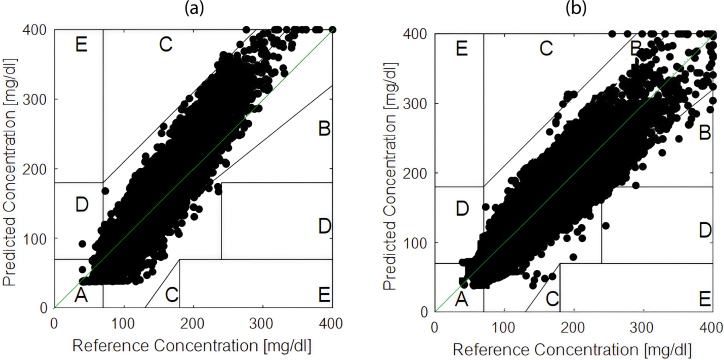
Clarke’s Error Grid analysis comparing the transmitter’s glucose measurements to the CGM reference. (a) corresponds to the non-calibrated c-rtCGM configuration, showing raw NFC-based measurements. (b) depicts the calibrated c-rtCGM configuration, where daily user-specific adjustments were applied, resulting in improved accuracy. Data points located within Zones A and B are considered clinically acceptable.

The lowest performance was a MARD of 13.38% for BG inferior to 70 mg/dl in calibrated devices, and for the range of 250–300 mg/dl for uncalibrated devices with a MARD of 14.19%. Fig. 11 illustrates Clarke’s Error Grid analysis comparing the daily calibrated and uncalibrated devices. The percentage of data points falling in zone A and B reaches 97% (as described in Table 3), indicating that the developed transmitter can be considered for clinical applications.

**Fig. 12:**
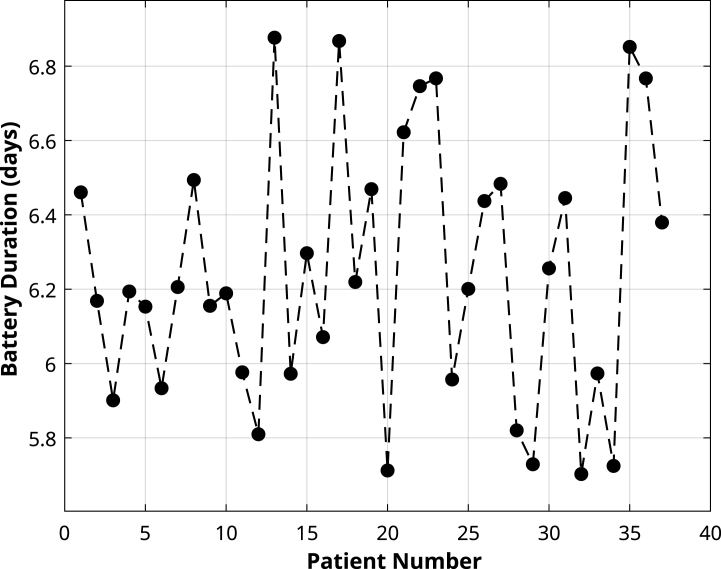
Battery life total duration discriminated for each participant. The mean value was 6.3 ± 0.6 days.

The battery life of the transmitter was evaluated throughout the 37 tests conducted with patients, showing variations in discharge time. The results, presented in Fig. 12, indicate that the minimum recorded duration was approximately 5.7 days, while the maximum reached 6.9 days, with an average of 6.3 days.

**Table 3:** Percentages in zones according to data interval.

Data interval (h)	Uncalibrated	Calibrated
Zone A (%)	83.06	91.65
Zone B (%)	12.68	6.76
Zone C (%)	3.04	1.23
Zone D (%)	1.22	0.36
Zone E (%)	0.00	0.00

Additionally, the data transmission success rate was also evaluated during the trial. The transmission connection event was launched every five minutes, and four attempts were allowed. If the system cannot retrieve the information after that, a connection failure is recorded. System disconnection may be due to various causes, including physical disconnection of the transmitter from the sensor, disconnection of the sensor from the patient, and communication errors within the system. During the trial, the most common disconnection error was the third one. In one patient, the sensor was replaced because of a failure, but the event was solved in three hours.

The results obtained were 66525 connection events, 2316 connection failures, and a data transmission success rate of 96.52%, representing 5543.8 h of successful connections, and only 193 h of disconnection.

**Fig. 13:**
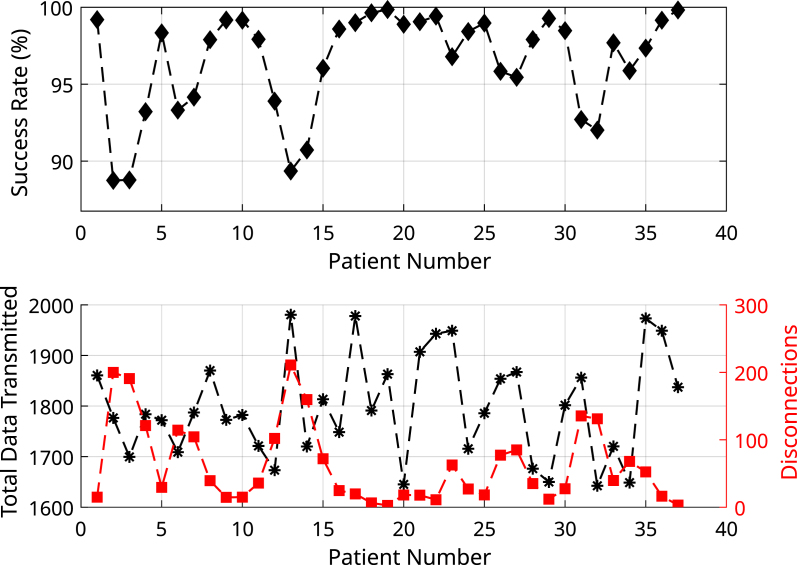
The first graph represents the success rate of connection stability for each of the 37 tests conducted with patients. The second graph displays the total amount of data transmitted by each patient, along with the number of disconnections recorded. To facilitate comparative visualization, the data has been scaled and represented in the same figure.

In Fig. 13, the success rate of data transmission, the total data transmitted, and the number of failures discriminated for each participant are depicted, demonstrating a high communication stability of the transmitter.

### 8.4 Discussion

The validation of the c-rtCGM transmitter demonstrated accuracy comparable to the CGM sensor system (FSL), particularly in critical glycemic ranges. The analysis of MARD revealed that, under daily calibration, the device achieved an overall value of 8.79%, with outstanding performance in hyperglycemic ranges (<180 mg/dL), where MARD decreased to 7.54%. These results suggest no relevant clinical differences between the developed transmitter systems and the FSL system monitoring, and coincide with the results achieved in [22].

However, in the hypoglycemic range (<70 mg/dL), MARD increased to 11.83% even with a daily calibration, a phenomenon also documented for the FSL (13.3% in studies such as Olfsdottir in [9]). This finding highlights an inherent challenge of interstitial glucose-based technologies: signal attenuation in hypoglycemia due to physiological delays and lower blood–interstitial correlation in this range.

This behavior aligns with observations from Gómez Medina et al. [24], who reported a MARD of 23.72% in hypoglycemia for open-source and auto-calibrated systems (e.g., xDrip+MM).

In this situation, it is recommended to take a capillary measurement when readings fall below 60 mg/dL and recalibrate the system with that reading before taking any action. To mitigate the health impacts on patients due to measurement errors in this zone, a safeguard is typically implemented in insulin delivery systems, which involves shutting down the pump if the trend falls below 70 mg/dL.

The clinical significance of the results shown in Table 1, Table 2 lies in their direct impact on therapeutic decision-making. For instance, under stable conditions (fluctuations <2 mg/dL/min), the MARD of the calibrated c-rtCGM (8.8%) falls below the 10% threshold recommended by the ISO 15197:2015 standard for self-monitoring devices, validating its use in AIDS where real-time accuracy is critical. However, in the absence of calibration, MARD increased to 12.18%, peaking at 14.19% in the 250-300 mg/dL range, highlighting the system’s dependence on periodic adjustments to maintain its performance.

Furthermore, the validation of the developed c-rtCGM transmitter demonstrated accuracy superior to previously evaluated systems. In the study by Villa-Tamayo et al. [23], which assessed the xDrip+MM system, an overall MARD of 12.06% was reported when using automatic calibrations, further emphasizing the superiority of the daily manual calibration approach implemented in the c-rtCGM. This also suggests that the calibration model is not working appropriately in xdrip+, since the hardware captures the same raw values.

The analysis using the Clarke Error Grid provided an additional clinical perspective. In calibrated devices, 91.65% of measurements were located in Zone A (clinically irrelevant differences) and 6.76% in Zone B (errors that would not alter treatment), totaling 98.41% clinically safe readings. This performance surpasses that reported for the FSL in studies such as Bailey et al. [2], where 86.7% of measurements were in Zone A.

Moreover, the calibrated system demonstrated superior accuracy compared to previous studies, achieving 91.65% in Zone A, significantly outperforming the 84.71% reported for Xdrip+MM by Villa et al. [23].

In contrast, non-calibrated devices recorded 83.06% of measurements in Zone A and 12.68% in Zone B, totaling 95.74%. Most deviations occurred in ranges above 180 mg/dL, suggesting a systematic bias linked to the static linear mapping function. While these discrepancies do not pose severe risks, they may lead to underestimating prolonged hyperglycemia, a critical factor in developing microvascular complications.

In terms of operational stability, the transmitter demonstrated a data transmission success rate of 96.52% over 66525 attempted connections, with only 2316 recorded failures. The average autonomy of 6.3 days per charge (range: 5.7–6.9 days) allows continuous use throughout the FSL sensor’s lifespan (14 days), requiring only one intermediate recharge.

Despite the promising results, the study presented methodological limitations that must be contextualized: a non-randomized sequential design allowing potential temporal biases, calibrations based on fixed schedules, and exclusion of specific populations.

The development of a low-cost, open-source customized transmitter represents a significant advancement in low-cost and open-source AIDS. Unlike commercial transmitters and sensors designed for continuous glucose monitoring, our ESP32-based transmitter offers flexibility, expandability, and enhanced integration with advanced control algorithms. However, using this transmitter in an AIDS system will depend on whether the FSL system encrypts raw data. If that happens, the transmitter can serve other devices that use the ISO/IEC 15693, ISO/IEC 14443-3, and ISO/IEC 18000-3M-1 protocols, as the chip CR95HF-IC supports these protocols.

### 8.5 Conclusion

The NFC-to-BLE transmitter for continuous real-time glucose monitoring (c-rtCGM) of isCGMs developed is a viable option in terms of reliability since it performs similarly to FSL system, guaranteeing high data transmission, to be used in a closed-loop system to regulate blood glucose in persons with type 1 diabetes.

The results of validation, in which the transmitter system was compared with the FSL system, confirm that the transmitter achieves high accuracy (a MARD of 8.8%) when one daily calibration is performed, is reliable according to the rate of transmission, and the duration of the battery life, showing that the hardware works adequately. The calibration method for converting raw values to blood glucose levels can be improved, suggesting calibrations when a maximum deviation is reached or developing a prediction algorithm with low dependence on calibration.

The advantages of this ESP32-based transmitter over commercial ones (as the MM), is its cost (53.42 USD lower than the 300 USD of MM) and software flexibility, allowing the incorporation of other features into the AIDS systems, such as direct modification of parameters in firmware, event-based control and activation/deactivation of other devices in the loop, prediction, filtering, and direct connection with cloud systems to share and safe data.

As this transmitter is envisioned for experimental and research studies, it is suggested to improve the housing using medical-grade materials (as the PLA Biocompatible ISO 10993-5/10 certified) to avoid possible skin irritations if it is intended for other uses.

One disadvantage of this hardware and its application to diabetes is that it depends on the commercial FSL sensor and its policies about data encryption. However, it can be adapted to other devices using the same communication protocols supported by the chip CR95HF-IC. Specifically, these protocols are ISO/IEC 15693, ISO/IEC 14443-3, and ISO/IEC 18000-3M-1. However, this option has not been explored since our primary motivation is in diabetes applications.

As future work, it is envisioned to integrate a customized, low-cost, and open-source insulin pump to generate a low-cost AIDS that can be used to regulate blood glucose in persons with type 1 diabetes. However, several important tasks are related to improving the raw glucose conversion using calibration methods, safeguards, and alarms to alert the person, as well as ensuring the cybersecurity of the system to prevent external intruders from compromising the person’s health through this system.

**Abbreviations**

• BG: blood glucose
• CGM: continuous glucose monitor
• c-rtCGM: continuous real-time glucose monitor
• CSII: continuous subcutaneous insulin infusion
• IQR: interquartile range
• isCGM: intermittent scanned glucose monitor
• MARD: mean absolute relative difference
• MDI: multiple daily injections
• RoC: rate of change
• SMBG: self-monitored blood glucose
• TAR: time above range
• TBR: time below range
• TIR: time in range.

## CRediT authorship contribution statement

**Pablo S. Rivadeneira:** Writing – original draft, Validation, Supervision, Resources, Project administration, Methodology, Funding acquisition, Formal analysis, Conceptualization. **Alejandro Mira:** Writing – original draft, Visualization, Software, Methodology, Data curation.

## Declaration of competing interest

We declare no conflict of interest.
